# Intra- and inter-biosynthetic gene cluster allelic variation as drivers of chemical diversification in *Streptomyces*

**DOI:** 10.1042/EBC20250013

**Published:** 2026-08-19

**Authors:** Golsa Nayeb G. Hosseini, Francisco Barona-Gómez

**Affiliations:** Evolution of Microbial Chemodiversity Laboratory, Institute of Biology Leiden University, Sylviusweg 72, 2333 BE, Leiden, The Netherlands

**Keywords:** allelic variation, biosynthetic gene cluster, chemodiversity, evolution, heterogeneity, Streptomyces

## Abstract

In this essay, we postulate that biosynthetic gene clusters (BGCs) chemical diversity arises in *Streptomyces* cultures from allelic variation both within a single BGC—intra-BGC, and across homologous BGCs—inter-BGC. These layers of genetic diversification reshape pathway architecture, modulate catalytic efficiency, and redefine substrate channeling, ultimately expanding the repertoire of specialized metabolites produced by *Streptomyces*. On one hand, previous metabolic analyses enabled by the one strain–many compounds framework have provided a comprehensive view of such chemical diversity, while enzyme promiscuity has emerged as a key mechanistic driver contributing to this chemical diversification generating expanded suites of structural analogs. On the other hand, large comparative genomics datasets and phylogenomics have revealed recurrent patterns of enzyme modular rearrangement, domain-level substitutions, and regulatory rewiring that underpin vertical metabolite divergence. Together, the interplay of BGC allelic heterogeneity, pathway modularity, and catalytic promiscuity forms a multilayered evolutionary strategy that fuels the emergence of novel chemical space—derived from a single BGC—in *Streptomyces* cultures.

## Introduction

The extraordinary chemical diversity characteristic of *Streptomyces* species emerges from a multilayered interplay of genetic architecture, enzymatic plasticity, and environmental modulation [1,2]. As prolific producers of specialized metabolites, *Streptomyces* bacteria harbor numerous biosynthetic gene clusters (BGCs) that encode the enzymatic machinery responsible for generating structurally complex and biologically potent compounds. While historically viewed as relatively static genomic units, BGCs are now understood to be dynamic entities whose output is shaped by both intrinsic molecular features and extrinsic ecological cues [3,4]. Notably, a single BGC can yield a spectrum of structurally related metabolites [5], demonstrating that the relationship between genotype and chemical phenotype is not linear but deeply dependent on phylogenetic, geographical [6–9] and regulatory context [10,11]. A major contributor to this metabolic breadth is enzyme promiscuity, a phenomenon in which secondary metabolism biosynthetic enzymes—such as modular polyketide synthases (PKSs) and nonribosomal peptide synthetases (NRPSs)—tolerate diverse substrates or catalyze alternative reaction chemistries [12].

The OSMAC (One Strain–Many Compounds) framework, originally introduced by Bode and co-workers [13] and expanded by subsequent studies (e.g. [14] and Table 1), has established that altering cultivation parameters—such as nutrient composition, oxygen availability, stressors, or physical culture format—can activate silent or weakly expressed BGCs. Recent studies highlighted that such condition-dependent activation reveals cryptic metabolites that remain undetectable under standard laboratory conditions. This framework underscores that the metabolic repertoire of *Streptomyces* is far richer than what canonical laboratory culturing typically uncovers, and that environmental signals act as key regulators of chemical output [1,2]. Interestingly, this approach unveils a wealth of chemical diversity encoded for and produced by a single BGC and biosynthetic pathway, analytically detectable as molecular networks consisting of a large number of analogs or related metabolites [5].

**Table 1:** Genetic and chemical mechanisms underlying *Streptomyces* chemical diversity

*Streptomyces*, metabolite	Genetic Mechanism	Chemical mechanism	OSMAC^a^	Enzyme promiscuity^b^	Reference
*Streptomyces coelicolor*, actinorhodin	Gene duplication and divergence	Duplicate biosynthetic genes mutate and gain new functions, creating chemical analogues.	Yes	Yes	[23]
*Streptomyces felleus*, pikromycin	PKS/NRPS module or domain swapping	Recombination swaps catalytic domains and modules, altering chain length and functional groups.	No	Yes	[24]
*Streptomyces coelicolor*, actinorhodin	Gain and loss of tailoring enzyme genes	Addition or removal of glycosyltransferase creates chemical analogues.	No	Yes	[25]
*Streptomyces* sp. IB2014/011-12, alpiniamide	Alternative enzyme module gene expression	Domain/module skipping and double bond migration events create chemical diversity.	No	Yes	[26]
*Streptomyces clavuligerus*, tunicamycin	Regulatory mutations	Changes in transcription factors modify activity and metabolic output.	Yes	No	[27]
*Streptomyces albus* J1074, candicidin	Formation of superclusters of BGC	Fusion of two or more BGCs creates hybrid pathways and metabolites.	No	Yes	[28]
*Streptomyces fradiae*, neomycin	Point mutations in biosynthetic domains	Single-point mutations alter substrate specificity or enzymatic activity.	No	Yes	[29]
*Streptomyces arenae* DSM40737, naphthocyclinone	Systems-level integration of enzyme function	Genomic changes lead to different metabolite outputs. Intra-BGC allelic variations?	Yes	Yes	[30]
*Streptomyces* sp. 1A1-3, aminopolycarboxylate	Population level dynamics	Intra- and Inter-BGC allelic variation driving chemical diversity?	No	No	[9]

^a^Refers to evidence related to OSMAC ^b^ or enzyme promiscuity approaches to increase chemical diversity, as interpreted by us but in many cases also by the authors. The selected examples aim at highlighting the point made and not to provide a comprehensive list of cases found in *Streptomyces*.

Enzyme promiscuity and environmental triggers seem therefore to account for the breadth of adaptive chemical diversity across *Streptomyces*. In this context, *Streptomyces* genetic heterogeneity at the population level hints toward allelic variation within and between homologous BGCs as a major evolutionary engine of metabolic divergence [15,16]. BGC allelic variation in sympatric *Streptomyces* strains or species [9,11,17] are expected therefore to modulate detectable shifts in metabolite structure. Subtle sequence differences—ranging from single nucleotide polymorphisms (SNPs) to localized indels or domain reshuffling—can indeed shape chemical diversity. For instance, SNPs within the peucemycin BGC of *Streptomyces peucetius* have been found to correlate with changes in metabolite bioactivity and structural modification patterns [18]. Such dynamic BGC evolution has resulted in extensive chemical diversification, with closely related *Streptomyces* strains often harboring unique clusters that encode structurally distinct metabolites but also conserved clusters that direct the synthesis of different metabolites [19].

The abovementioned observations situate chemical diversity in *Streptomyces* as an emergent property of an intricately interconnected system: enzyme promiscuity provides biochemical elasticity; and environmental modulation unveils latent metabolic potential. On top of this, here we argue that population level allelic variation—both within BGCs, i.e. intra-BGC, and across BGCs, i.e. inter-BGCs—drives, in addition to long-term evolutionary vertical diversification, the chemical diversity commonly found in *Streptomyces* cultures and environmentally sympatric strains. This triad of factors forms a foundation for the understanding of the evolutionary drivers guiding the biosynthesis of specialized metabolites by *Streptomyces* species, as postulated by the dynamic chemical matrix (DCM) evolutionary hypothesis [3,20]. The DCME framework also overlaps with the concept of underground metabolism, which refers to metabolic networks as the consequence of the main biochemical conversions, but also the weak promiscuous enzyme activities, metabolic side reactions that operate outside canonical pathways and nonenzymatic spontaneous reactions. Such reactions can generate noncanonical or damaged metabolites, some of which require metabolite repair or detoxification, whereas others may provide raw material for metabolic innovation and chemical diversification [21,22].

In this essay, we first provide examples of *Streptomyces* chemical diversity unveiled by OSMAC and enzyme promiscuity experiments, and relate these metabolite examples to the potential evolutionary and chemical mechanisms underlying these observations (Table 1). These examples show several cases of synergistic contribution of both mechanisms toward the apparent chemical diversity; however, they also highlight that unappreciated mechanisms are in place. To gain deeper insights into this, we adopt *Streptomyces globisporus* SCSIO LCY30 [14] and perform a metanalysis of the chemical diversity of this strain using Global Natural Products Social (GNPS) molecular-networking, leading us to postulate the important and poorly understood role of intra- and inter-BGC allelic variation.

## Evolutionary origins of BGCs and their chemical diversity

Table 1 summarizes core genetic and chemical mechanisms responsible for generating the extraordinary chemical diversity observed across *Streptomyces* specialized metabolites. These mechanisms—including gene duplication, horizontal gene transfer, PKS/NRPS recombination, tailoring enzyme variation, BGC rearrangement, regulatory evolution, supercluster formation, and domain-level mutations—collectively shape the complexity and variability of BGCs and their chemical products. However, even if comprehensive, these genetic and chemical mechanisms only provide an explanation of how chemical diversity arises in the long term, but not within *Streptomyces* cultures expected to be monoclonal and homogeneous. To a lesser extent this would also apply to sympatric *Streptomyces* populations. Thus, while these previous studies have uncovered the molecular basis of the diversification processes driving the emergence of novel metabolic architectures and chemical scaffolds, they fail to explain the chemical diversity produced by one single BGC and its cognate pathway.

In an attempt to address this knowledge gap about the evolutionary origin and dynamics of BGCs, we have previously proposed the DCM evolutionary hypothesis [3,20]. The DCM framework posits that specialized metabolism arises from the recruitment and expansion of promiscuous enzyme families originally involved in central metabolism, which are gradually assembled into new functional clusters through gene duplication, horizontal gene transfer, and recombination [3,20]. These genetic mechanisms, together with point mutations, are a major driver of chemical diversity in *Streptomyces* (Table 1). *Streptomyces* chromosomal instability can also move or replace entire BGCs between closely related strains and species, creating mosaic genomes and strong differences in metabolite production [31,32]. Within a single filamentous colony, this may also generate subclonal variation, leading to localized differences in chemistry. In natural environments, frequent contact between cells likely promotes continuous gene exchange, linking strain-level and population-level diversity [9].

At the enzyme level, gene duplication or expansion provides the raw material for innovation, allowing paralogs and analogs to evolve novel catalytic functions. At the genomic level, recombination and modular rearrangement within and between clusters further enhance chemical diversity by shuffling enzymatic domains, tailoring genes, and regulatory elements. According to this model, BGCs, as dynamic and modular entities that emerge, diversify, and disappear through a continuum of genetic and ecological processes, are constantly under both positive and negative selection ensuring selection in the long term of chemical diversity per se irrespective of the long term selection of specific or final natural products.

**Figure 1:**
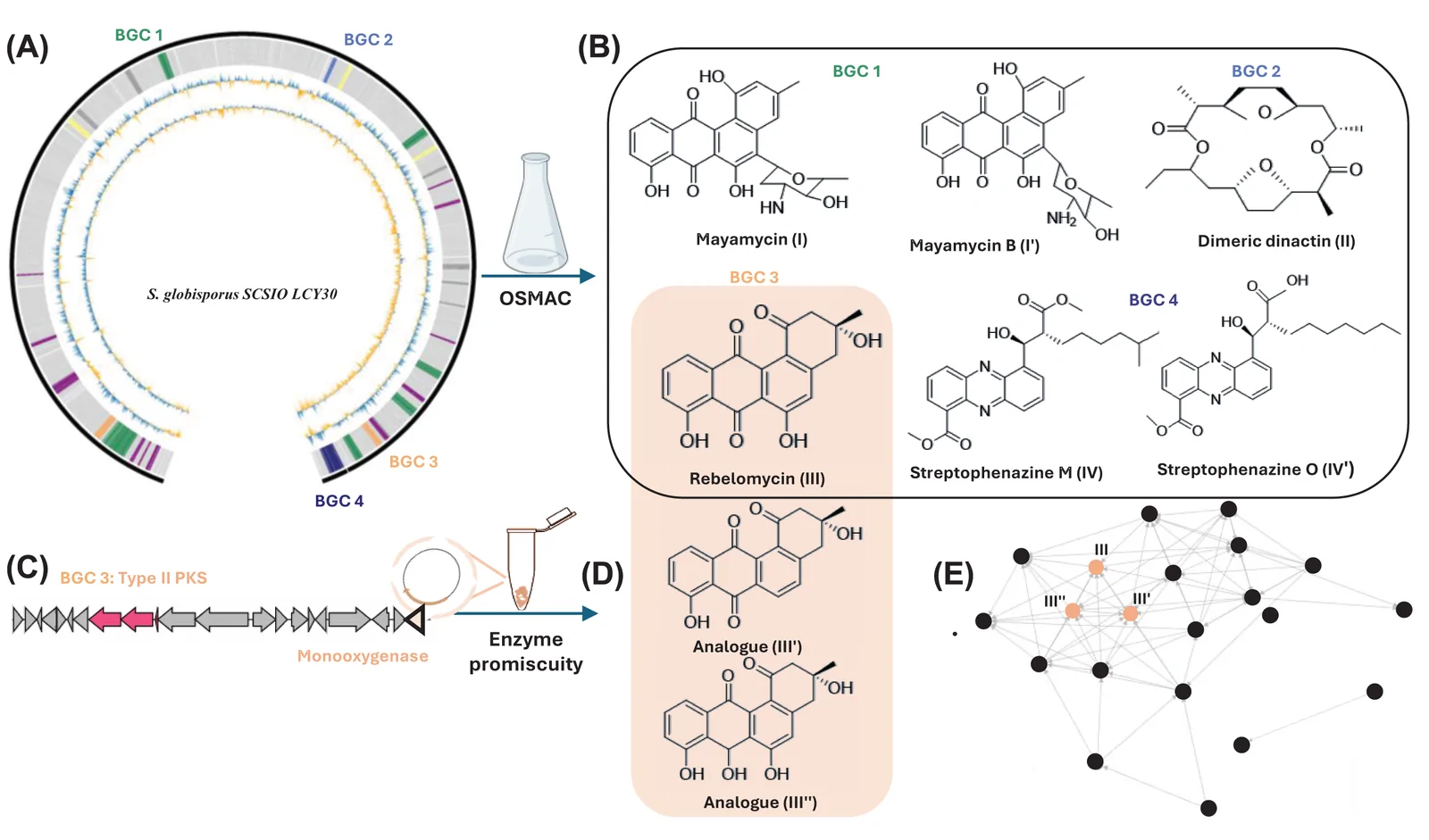
OSMAC and enzyme promiscuity driving chemical diversification in *S. globisporus* SCSIO LCY30 (**A**) antiSMASH analysis of the complete genome reveals numerous BGCs including four PKS BGCs (BGC 1–4) that encode type I, II, and III PKSs. (**B**) Cultivation under varied media and regulatory states (OSMAC) activates different clusters from the same genome leading to expanded chemical diversity including different analogues. (**C**) Organization of BGC 3 highlighting the PKS genes (pink) and the tailoring monooxygenase (light beige) cloned and purified for *in vitro* enzyme promiscuity experiments. (**D**) Chemoenzymatic diversification using the monooxygenase, and complementarity with OSMAC (shaded box). The structural analogs depicted in panels (B) and (D) are mayamycin (I), mayamycin B (I″), dimeric dinactin (II), rebelomycin (III), streptophenazine M (IV), and streptophenazine O (IV′). (**E**) GNPS molecular network integrating chemical diversity highlighting analogs (black nodes) that are not accounted for by neither of OSMAC or enzyme promiscuity. The network is used in a hypothesis-generating and illustrative manner to visualize chemical relatedness among detected metabolites, rather than to assign definitive biosynthetic origins to every node.

**Figure 2:**
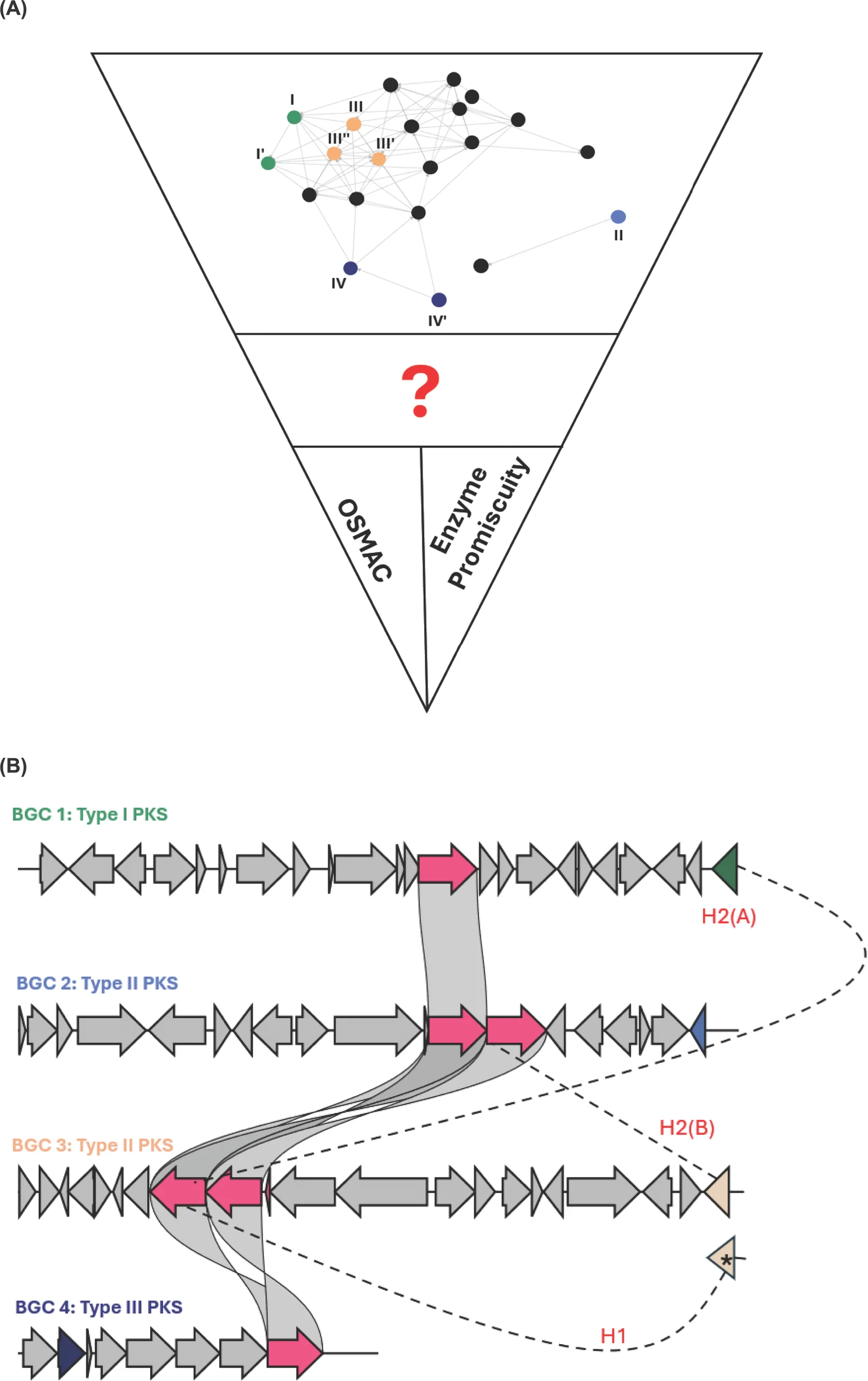
Conceptual framework for hidden chemical diversity via intra- and inter-BGC allelic variation (**A**) Inverted pyramid model illustrating how currently recognized strategies—OSMAC and enzyme promiscuity—capture only part of the potential metabolic space. The central red question mark highlights new mechanisms, which could be intra- and inter-BGC allelic variation. Network nodes represent metabolite families, with colored nodes corresponding to compounds linked to distinct PKS BGCs in *S. globisporus* SCSIO LCY30 as shown in Figure 1. (**B**) Comparative gene order of four PKS BGCs showing limited synteny, yet homologous PKS genes (pink arrows). The hypothesized metabolic diversification routes (H1, H2) are shown with dashed lines. For H2, two possibilities are provided: a monooxygenase from H1 (H2(B)), and a glycosyltransferase from BGC1 (H2(A)). These evolutionary scenarios implicate inter- and intra-BGC allelic variations as drivers of chemical diversification in *Streptomyces*. Genes highlighted in pink encode for KS-like condensation enzymes, and a such can interact with several enzyme domains and substrates.

Together, these mechanisms underpin the evolutionary origin and dynamics of BGCs, as envisioned in the DCM evolutionary hypothesis, revealing how continuous molecular innovation and genomic reorganization drive the emergence of new metabolic traits across microbial lineages [3,20]. However, the DCM model does not explicitly refers to the chemical diversity encountered in *Streptomyces* cultures driven by OSMAC and enzyme promiscuity approaches (Figure 1). As introduced, we adopt *S. globisporus* SCSIO LCY3 [14], a producer of several polyketides—involving types I, II, and III PKS—as a case study to highlight the interrelationship of these two factors. Our metanalysis of the chemical diversity of this strain using GNPS molecular-networking (Figure 1) highlights that chemical diversity cannot be explained solely on the basis of these two factors, leading us to postulate the important and poorly understood role of intra- and inter-BGC allelic variation (Figure 2).

## Are OSMAC and enzyme promiscuity the whole story?

As introduced, the remarkable chemical diversity observed in *Streptomyces* arises from two key factors: the OSMAC framework and enzyme promiscuity. In recent times, these two features have become apparent due to enhanced modern analytical metabolomics tools such as feature-based molecular-networking and MS languages [33]. Molecular-networking platforms such as GNPS have become indispensable tools for visualizing and analyzing the resulting metabolomic complexity of *Streptomyces* [34]. By clustering compounds based on MS/MS fragmentation similarity, molecular networking enables the identification of structurally related metabolites, facilitating dereplication, and discovery of novel analogues [35]. Induced pathway activation, enzyme-driven diversification, and network-based metabolome mapping form a synergistic triad that underpins modern natural product discovery [36]. A good example of this situation, and used herein to demonstrate how the OSMAC approach and enzyme promiscuity can effectively unlock *Streptomyces* chemical diversity, is provided by *S. globisporus* SCSIO LCY30 [14].

The authors mining *S. globisporus* SCSIO LCY30 successfully exploited variations in culture media, nutrient composition, and adsorbent addition to activate and detect the metabolites of previously silent BGCs [14]. The strain produced a wide range of metabolites—including angucycline-type polyketides, phenazine derivatives, and macrolides—each representing distinct biosynthetic families that collectively highlight the strain’s metabolic plasticity [14]. Figure 1 illustrates an integrated overview of the BGCs encoded by the genome of SCSIO LCY30 (panels (A) and (C)), next to the results obtained after an OSMAC-based (panel (B)) and enzyme promiscuity (panel (D)) exploration. The biosynthetic potential of SCSIO LCY30 includes four PKS BGCs with different locations across the chromosome: BGC1 (Type I PKS), BGC2 (Type II PKS), BGC3 (Type II PKS), and BGC4 (Type III PKS). Panel (B) shows the chemical structures of the metabolites arising from these BGCs, namely, mayamycin I and mayamycin B (I′, I), which involves a glycosyltransferase-mediated reaction (dark green gene, BGC1); dimeric dinactin (II) derived from nonactate synthase (light blue, BGC2); rebelomycin (III) generated by a monooxygenase enzyme (light pink gene, BGC3, also shown in panel (C)); and streptophenazine M and O (IV, IV′), which implicates a methyltransferase during its synthesis (dark-blue gene, BGC4). Panel (D) presents the promiscuously synthesized metabolites by the monooxygenase responsible for the formation of rebelomycin analogues (III′, III″) *in vitro* enzyme assays. Together, this chemical diversity is shown in panel (E) by means of an integrated molecular network generated with both OSMAC and enzyme promiscuity metabolomic datasets, where compounds III, III′, and III″ cluster together with other related metabolites that do not show up in neither the OSMAC nor the enzyme promiscuity datasets.

## Intra- and inter-BGC allelic variations as drivers of chemical diversity in *Streptomyces* populations

While these observations reveal the complementarity between OSMAC and enzyme promiscuity, they also speaks to the chemical diversity often encountered in *Streptomyces* cultures that is not accounted for by these well-established mechanisms. This is further developed in Figure 2, which postulates that intra- and inter-BGC genetic variability could account for additional chemical diversity. An inverted pyramid (panel (A)) is used to depict how enzyme promiscuity and OSMAC require other unappreciated elements in completing the foundations for this chemical diversity (shown with an interrogation mark). The postulated intra- and inter-BGC genetic variability (panel (B)) is captured by the comparative gene order and sequence similarity of the four PKS BGCs of Figure 1, showing limited synteny yet homologous PKS genes (pink arrows). The hypothesized diversification routes (H1, H2) are shown with dashed lines, highlighting the potential for genetic recombination or evolutionary reshuffling among clusters in the long term, but also of the potential of biosynthetic enzymes interacting stochastically within and across BGCs at any given point on a *Streptomyces* culture.

Interesting antecedents to the possibility of metabolic interactions driven by BGC allelic variation at the culture (population) level, are the well-documented cases in which enzymes from primary metabolism are directly recruited into specialized metabolic pathways. Such recruitments highlight the evolutionary plasticity of core metabolic enzymes, which can be co-opted to generate chemical diversity beyond their original physiological roles [37]. In the context of *Streptomyces* cultures, when primary and/or secondary metabolic pathways are perturbed (e.g. through a gene knockout), crosstalk between enzymes and intermediates can emerge, restoring the ability of the *Streptomyces* strain to produce the corresponding metabolites. Examples of this type of inter-BGC crosstalk are provided by chorismate-utilizing enzymes involved in the synthesis of several secondary and central metabolites [38,39] and the nucleoside antibiotics using L-histidine biosynthetic enzymes [40].

The integration of OSMAC and enzyme promiscuity with intra- and inter-BGC allelic variation, whose consequences are expected to be governed by population-level dynamics, could collectively generate and broaden the chemical space reflected in the molecular network of Figure 2. It is worthwhile to note that metabolites I–IV group into distinct but interconnected families and therefore could be generated by either inter- or intra-BGC allelic variations to produce different metabolites with related scaffolds in *S. globisporus* SCSIO LCY30. Interestingly, by the combination of these two genetically encoded possibilities, the inter- and intra-BGC allelic variation could act exponentially explaining the vast chemical diversity produced by *Streptomyces*. This possibility is capture by the two complementary evolutionary hypotheses H1 and H2, as shown with dashed lines in Figure 2 and detailed next, in line with a new paradigm of one BGC—many metabolites.

### H1: Intra-BGC allelic variation

The first scenario involves noncore modular biosynthetic enzymes (i.e. PKS and NRPS genes) within a BGC functionally interacting with the product of tailoring enzymes’ genes located within the same cluster. In this example, a monooxygenase enzyme variant may modify the product of the PKS of its own BGC, leading to the production of structurally distinct metabolites. Intra-BGC allelic variation represents a mechanism by which localized genetic changes can fine-tune metabolite output in response to the dynamics of the population (even in ‘monoclonal’ cultures). This would allow a single BGC to generate chemical diversity without requiring large-scale genomic rearrangements, providing a rapid and efficient means of producing structurally diverse compounds.

### H2: Inter-BGC allelic variation

The second scenario implicates core genes functionally interacting with core biosynthetic genes across distinct BGCs. In this scenario, tailoring enzymes such as the monooxygenase (example B) and the glucosyltransferase (example A) are not limited to their own BGC but can interact with the PKSs encoded elsewhere in the genome. Inter-BGC allelic variation exemplifies a genome-wide mechanism for expanding chemical diversity at the population level. By enabling enzymes to act across the enzymes of multiple clusters, this mechanism can generate novel metabolites without the need to evolve entirely new biosynthetic pathways. Inter-BGC interactions serve as a potent amplifier of chemical innovation, increasing the likelihood of producing metabolites with new structural and functional properties.

To further explore these hypotheses, the concept of the metabolon provides a useful mechanistic framework for understanding how such inter-BGC interactions may be physically realized in *Streptomyces*. One plausible model suggests that producing chemical diversity in *Streptomyces* populations is shaped by transient enzyme–enzyme assemblies, referred to as metabolons, in which sequential or functionally related enzymes physically associate through weak, noncovalent interactions [12,41].

In this context, additional nodes displaying distinct m/z values and unique LC–MS/MS fragmentation patterns, which do not correspond to known OSMAC-elicited molecules or enzyme-permissive variants, point to the existence of these other mechanisms. The existence of unidentified metabolites [7,9,17] within core BGCs suggests that our current understanding of BGC-driven chemical diversity is incomplete. Furthermore, genomic analysis of *Streptomyces* populations has revealed extensive variation in BGC gene content [32]. In silico studies have also predicted that BGCs are prone to recombination, leading to the emergence of novel biosynthetic pathways in microbial populations [42,43]. Importantly, the integration of genomic data with metabolomics has already identified several examples of noncanonical BGC interactions [17,44,45].

## Conclusion and perspectives

In this essay, we posit that the expansive chemical repertoire of *Streptomyces* secondary or specialized metabolism is fundamentally governed by genetic allelic variation manifesting across distinct organizational hierarchies of BGCs. We argue that genetic diversification within individual clusters (intra-BGC) remodels pathway architecture by modulating enzyme kinetics, substrate specificity, and catalytic flux, whereas diversification across homologous clusters (inter-BGC) orchestrates a reorganization of entire biosynthetic frameworks through pathway rewiring. These genomic perturbations do not act in isolation; rather, they are functionally potentiated by enzyme promiscuity, which instills biochemical plasticity into the pathway and enables the generation of expanded suites of structural analogs from shared scaffolds.

Looking forward, the systematic deconvolution of this genotype–chemotype relationship relies on bridging untargeted metabolomics with molecular-networking through the GNPS platform at the population level. Complementing this is Desorption Electrospray Ionization Mass Spectrometry (DESI–MS), a technology that has seen significant advancements regarding sensitivity and spatial resolution [46]. For future developments, DESI–MS/MS workflows should be advanced toward automation similar to LC–MS/MS, where ions are automatically selected through data-dependent or data-independent acquisition, fragmented systematically, and associated with retention time to improve annotation confidence. Such integration would enable high-throughput, unbiased fragmentation, reduce manual intervention, and support more robust and scalable molecular-networking.

**Figure 3:**
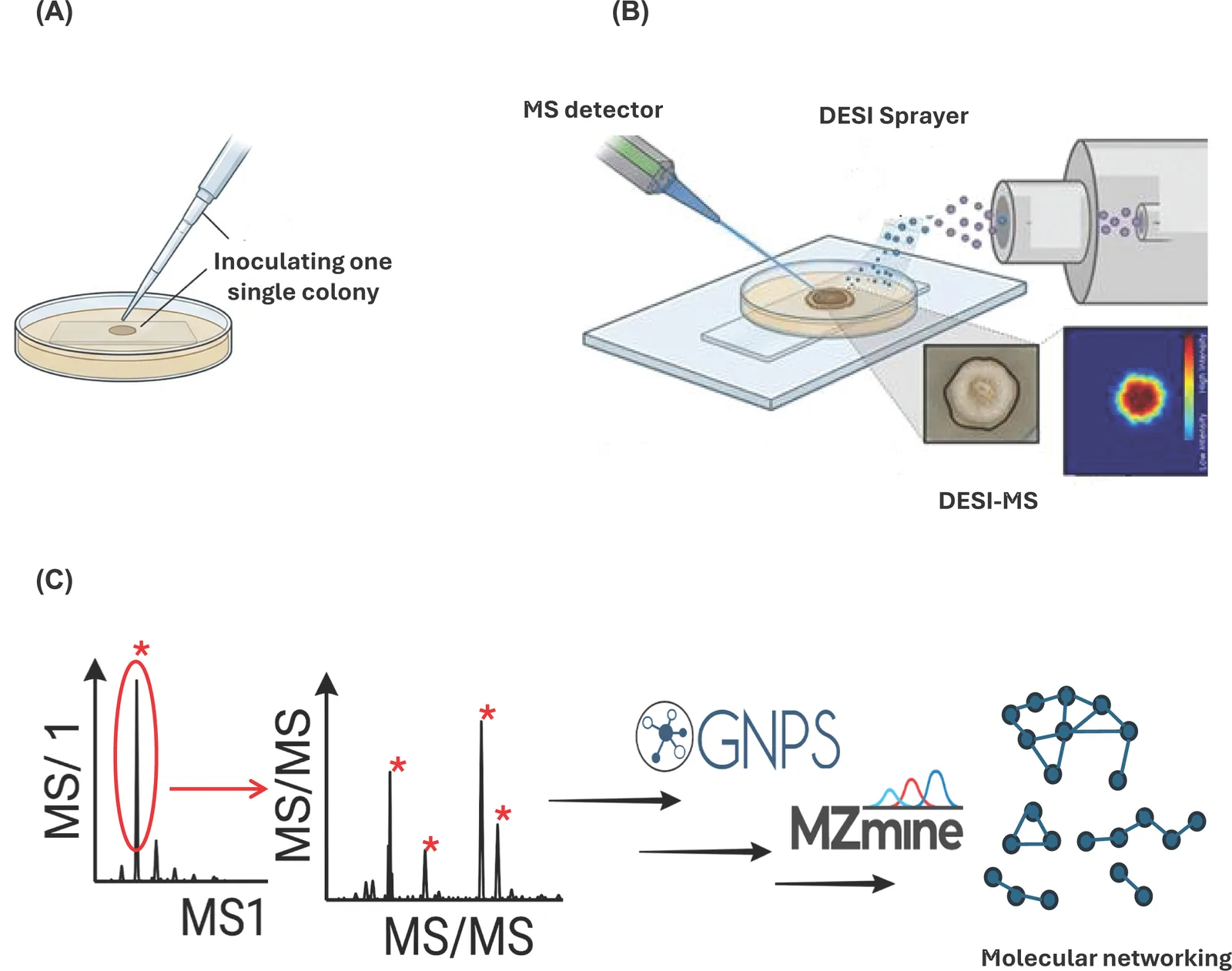
DESI–MS to reveal chemical diversity using molecular-networking with spatial resolution (**A,B**) Chemical diversity within a single microbial colony is visualized *in situ* using DESI–MS without extraction. (**C**) Currently, DESI–MS/MS is limited by the manual selection of MS1 ions for fragmentation (shown with red asterisks) needed for automated molecular-networking.

In the near future, chemical diversity within a single colony from one population can be directly visualized using DESI–MS, as illustrated in Figure 3. Here a single *Streptomyces* colony is analyzed *in situ* without sample preparation through spatial metabolomics at a sub-colony resolution level. As distinguishing isomers remains challenging integrating DESI–MS with spatial and single-cell transcriptomics would better link metabolite profiles to gene expression and allelic variation [47]. A key limitation of DESI–MS/MS is that, unlike LC–MS/MS, there is no retention time separation, making spectra deconvolution challenging. Currently, ions of interest must be manually selected and transferred to the MS/MS chamber, highlighting a major workflow bottleneck and knowledge gap. Future approaches therefore should aim at automatization of fragmentation and annotation of chemical diversity with spatial resolution.

By harnessing these future-ready capabilities of DESI–MS alongside large-scale comparative genomics, researchers can effectively correlate specific intra- and inter-BGC allelic variations with chemical diversity. Whether the metabolic diversity observed in *Streptomyces* cultures emerges from stochastic molecular events and/or from a deeply integrated evolutionary framework remains to be deciphered, but to answer this, the interplay of allelic heterogeneity, pathway modularity, and catalytic promiscuity needs to be addressed in the future.

## Summary

- Intra-BGC allelic variation results from mutations within a single cluster, subtly altering enzyme function and product structure, whereas inter-BGC allelic variation arises from homologous enzymes across multiple clusters in the same genome, collectively expanding substrate scope and chemical diversity during growth of *Streptomyces* populations.
- Interplay between these allelic variants increases the likelihood of cross-pathway interactions, where enzymes from different clusters act on shared or related intermediates, further expanding the chemical diversity of *Streptomyces* populations.
- Research must couple high-fidelity long-read sequencing with untargeted imagining metabolomics to systematically map specific allelic polymorphisms to distinct chemical outputs, thereby establishing a direct link between genotype and chemotype.

## Open Access

This article has been published open access under our Subscribe to Open programme, made possible through the support of our subscribing institutions, learn more here: https://portlandpress.com/pages/open_access_options_and_prices#conditional.

## Abbreviations

BGC: Biosynthetic gene cluster
DCM: Dynamic chemical matrix
DESI–MS: Desorption electrospray ionization mass spectrometry
GNPS: Global Natural Products Social
LC–MS: Liquid chromatography–mass spectrometry
MLST: Multi-locus sequence typing
MS/MS: Tandem mass spectrometry
NRPS: Nonribosomal peptide synthetases
OSMAC: One strain–many compounds
PKS: Polyketide synthases
SNP: Single nucleotide polymorphism

## References

[1] Cantu Morin L., Dekoninck K., Sridhar V., Disney-Mckeethen S., Proctor T., Eng A. et al. (2025) Why do filamentous actinomycetota produce such a vast array of specialized metabolites. Annu. Rev. Microbiol. 79, 753–772 10.1146/annurev-micro-060424-051257 40939134 PMC13117493

[2] Norte D., Avitia-Dominguez L. and Rozen D. (2025) Evolution and ecology of *Streptomyces*. Annu. Rev. Microbiol. 79, 383–403 10.1146/annurev-micro-051524-032254 40829782

[3] Chevrette M., Gutiérrez-García K., Selem-Mojica N., Aguilar-Martínez C., Yañez-Olvera A., Ramos-Aboites H. et al. (2020) Evolutionary dynamics of natural product biosynthesis in bacteria. Nat. Prod. Rep. 37, 566–599 10.1039/C9NP00048H 31822877

[4] Salamzade R. and Kalan L. (2025) Context matters: assessing the impacts of genomic background and ecology on microbial biosynthetic gene cluster evolution. mSystems 10, e01538–24 10.1128/msystems.01538-24 39992097 PMC11915812

[5] Linington R. (2025) An assessment of chemical diversity in microbial natural products. ACS Central Science 11, 1536–1545 10.1021/acscentsci.5c00804 41019115 PMC12464764

[6] Chase A., Sweeney D., Muskat M., Guillén-Matus D. and Jensen P. (2021) Vertical inheritance facilitates interspecies diversification in biosynthetic gene clusters and specialized metabolites. mBio 12, e02700–e02721 10.1128/mBio.02700-21 34809466 PMC8609351

[7] Hariharan J., Andam C. and Buckley D. (2025) Biogeographical and phylogenetic constraints on horizontal gene transfer and genome evolution *Streptomyces*. Microbiol Spectrum. 14, 41404872 10.1128/spectrum.02958-25 PMC12889140 41404872

[8] Creamer K., Castro-Falcón G., Ince E., Vasilat V., Vereau Gorbitz D., Demko A. et al. (2025) Taxonomic and biosynthetic diversity of the marine actinomycete *Salinispora* across spatial scales. Appl. Environ. Microbiol. 92, e02171–25 41358838 10.1128/aem.02171-25 PMC12838356

[9] Choufa C., Gascht P., Leblond H., Gauthier A., Vos M., Bontemps C. et al. (2024) Conjugation mediates large-scale chromosomal transfer *Streptomyces* driving diversification of antibiotic biosynthetic gene clusters. Mol. Biol. Evol. 41, msae236 10.1093/molbev/msae236 39506544 PMC11571958

[10] Augustijn H., van Nassauw D., Cernat S., Reitz Z., van Wezel G. and Medema M. (2025) Regulatory genes as beacons for discovery and prioritization of biosynthetic gene clusters *Streptomyces*. Biochemistry 64, 2877–2885 10.1021/acs.biochem.4c00711 40133269 PMC12224297

[11] Hoskisson P. and Fernández‐Martínez L. (2018) Regulation of specialised metabolites in actinobacteria—expanding the paradigms. Environ. Microbiol. Rep. 10, 231–238 10.1111/1758-2229.12629 29457705 PMC6001450

[12] Chevrette M., Hoskisson P. and Barona-Gómez F. (2020) Enzyme evolution in secondary metabolism. Comprehensive Natural Products III. 90–112, Elsevier, Amsterdam Chemistry and Biology. pp. 10.1016/B978-0-12-409547-2.14712-2

[13] Bode H., Bethe B., Höfs R. and Zeeck A. (2002) Big effects from small changes: Possible ways to explore nature’s chemical diversity. ChemBioChem 3, 619–627 10.1002/1439-7633(20020703)3:7<619::AID-CBIC619>3.0.CO;2-9 12324995

[14] Li Y., Gong N., Zhou L., Yang Z., Zhang H., Gu Y. et al. (2024) OSMAC-based discovery and biosynthetic gene clusters analysis of secondary metabolites from marine-derived *Streptomyces globisporus* SCSIO LCY30. Mar. Drugs. 22, 21 10.3390/md22010021 PMC10817512 38248647

[15] Hoskisson P., Barona-Gómez F. and Rozen D. (2024) Phenotypic heterogeneity *Streptomyces* colonies. Curr. Opin. Microbiol. 78, 102436 10.1016/j.mib.2024.102448 38447313

[16] Teng-Hern Tan L. (2025) Decoding multicellularity *Streptomyces:* single-cell technologies reveal the link between phenotypic heterogeneity and secondary metabolite production. Prog. Microbes Mol. Biol. 8, 10.36877//pmmb.a0000477

[17] Tidjani A., Lorenzi J., Toussaint M., van Dijk E., Naquin D., Lespinet O. et al. (2019) Massive gene flux drives genome diversity between sympatric *Streptomyces* conspecifics. mBio. 10, 01533–19 10.1128/mBio.01533-19 31481382 PMC6722414

[18] Pham V., Nguyen H., Nguyen C., Choi Y., Dhakal D., Kim T. et al. (2021) Identification and enhancing production of a novel macrolide compound in engineered: *Streptomyces* eucetius. RSC Adv. 11, 3168–3173 10.1039/D0RA06099B 35424263 PMC8693821

[19] Nguyen P., Ly L., Le M., Vuong B., Phan P. and Nguyen H. (2025) Unlocking *Streptomyces* biosynthetic gene clusters: bioelicitors, co-culture, and beyond. Syst. Microbiol. Biomanuf. 6, 1 10.1007/s43393-025-00397-6

[20] Barona-Gómez F., Chevrette M. and Hoskisson P. (2023) On the evolution of natural product biosynthesis. Adv. Microb. Physiol. 82, 309–349 10.1016/bs.ampbs.2023.05.001 37507161

[21] Rosenberg J. and Commichau F.M. (2019) Harnessing underground metabolism for pathway development. Trends Biotechnol. 37, 29–37 10.1016/j.tibtech.2018.08.001 30172385

[22] Notebaart R.A., Kintses B., Feist A.M. and Papp B. (2018) Underground metabolism: network-level perspective and biotechnological potential. Curr. Opin. Biotechnol. 49, 108–114 10.1016/j.copbio.2017.07.015 28837944

[23] Marshall A. and Carlson E. (2023) Metabolomics reveals a “trimeric” γ-actinorhodin from *Streptomyces coelicolor* M145. ChemBioChem 24, e202200650 10.1002/cbic.202200757 36729633

[24] Chemler J., Tripathi A., Hansen D., O’Neil-Johnson M., Williams R., Starks C. et al. (2015) Evolution of efficient modular polyketide synthases by homologous recombination. J. Am. Chem. Soc. 137, 10603–10609 10.1021/jacs.5b04842 26230368 PMC4666801

[25] Taguchi T., Yabe M., Odaki H., Shinozaki M., Metsä-Ketelä M., Arai T. et al. (2013) Biosynthetic conclusions from the functional dissection of oxygenases for biosynthesis of actinorhodin and related *Streptomyces* antibiotics. Chem. Biol. 20, 510–520 10.1016/j.chembiol.2013.03.007 23601640

[26] Paulus C., Rebets Y., Zapp J., Rückert C., Kalinowski J. and Luzhetskyy A. (2018) New alpiniamides from *Streptomyces* sp. IB2014/011-12 assembled by an unusual hybrid non-ribosomal peptide synthetase trans-AT polyketide synthase enzyme. Front. Microbiol. 9, 1959 10.3389/fmicb.2018.01959 30186270 PMC6113372

[27] Martínez-Burgo Y., Santos-Aberturas J., Rodríguez-García A., Barreales E., Tormo J., Truman A. et al. (2019) Activation of secondary metabolite gene clusters *Streptomyces clavuligerus* by the pimm regulator of *Streptomyces natalensis*. Front. Microbiol. 10, 580 10.3389/fmicb.2019.00580 30984130 PMC6448028

[28] González A., Rodríguez M., Braña A., Méndez C., Salas J. and Olano C. (2016) New insights into paulomycin biosynthesis pathway *Streptomyces* *albus* J1074 and generation of novel derivatives by combinatorial biosynthesis. Microb. Cell Fact. 15, 56 10.1186/s12934-016-0452-4 27001601 PMC4802897

[29] Li X., Yu F., Wang F., Wang S., Han R., Cheng Y. et al. (2022) Point mutation of V252 in neomycin C epimerase enlarges substrate-binding pocket and improves neomycin B accumulation *Streptomyces* *fradiae*. Bioresour. Bioprocess. 9, 114 10.1186/s40643-022-00613-4 38647873 PMC10991966

[30] Baral B., Matroodi S., Siitonen V., Thapa K., Akhgari A., Yamada K. et al. (2023) Co-factor independent oxidases ncnN and actVA-3 are involved in the dimerization of benzoisochromanequinone antibiotics in naphthocyclinone and actinorhodin biosynthesis. FEMS Microbiol. Lett. 370, fnad123 10.1093/femsle/fnad123 37989784 PMC10697411

[31] Bury-Moné S., Thibessard A., Lioy V. and Leblond P. (2023) Dynamics of the *Streptomyces* chromosome: chance and necessity. Trends Genet. 39, 873–887 10.1016/j.tig.2023.07.008 37679290

[32] Mohite O., Jørgensen T., Booth T., Charusanti P., Phaneuf P., Weber T. et al. (2025) Pangenome mining of the *Streptomyces* genus redefines species’ biosynthetic potential. Genome Biol. 26, 9 10.1186/s13059-024-03471-9 39810189 PMC11734326

[33] Damiani T., Jarmusch A., Aron A. et al. (2025) A universal language for finding mass spectrometry data patterns. Nat. Methods 22, 1247–1254 10.1038/s41592-025-02660-z 40355727 PMC12334354

[34] Nothias L., Petras D., Schmid R., Dührkop K., Rainer J., Sarvepalli A. et al. (2020) Feature-based molecular networking in the GNPS analysis environment. Nat. Methods 17, 905–908 10.1038/s41592-020-0933-6 32839597 PMC7885687

[35] Caraballo-Rodríguez A., Cumsille A., Magyari S., Taboada-Alquerque M., Behsaz B., Leão T. et al. (2025) The undiscovered natural product potential of actinomycetes. J. Antibiot. (Tokyo). 79, 80–92 10.1038/s41429-025-00876-x 41331045 PMC12834688

[36] Guler M., Krummenacher B., Hall T., Tandon M., Abrams J., Ravi S. et al. (2025) Identifying variants of molecules through database search of mass spectra. Nat. Comput. Sci. 5, 1227–1237 10.1038/s43588-025-00923-5 41326787

[37] Sélem-Mojica N., Aguilar C., Gutiérrez-García K., Martínez-Guerrero C. and Barona-Gómez F. (2019) EvoMining reveals the origin and fate of natural product biosynthetic enzymes. Microb. Genom. 5, e000260 10.1099/mgen.0.000260 30946645 PMC6939163

[38] Cano-Prieto C., Losada A., Braña A., Méndez C., Salas J. and Olano C. (2015) Crosstalk of nataxazole pathway with chorismate-derived ionophore biosynthesis pathways *Streptomyces* sp. Tü 6176. ChemBioChem 16, 1925–1932 10.1002/cbic.201500261 26083234

[39] Vining L. (1992) Secondary metabolism, inventive evolution and biochemical diversity—a review. World. J. Microbiol. Biotechnol. 8, 90–91 10.1007/BF02421504 24425657

[40] Rosas-Becerra L., Arredondo-Cruz A., Sélem-Mojica N. and Barona-Gómez F. (2026) Functional shifts of the dual-substrate phosphoribosyl isomerase PriA from primary to specialized metabolism in rare Actinomycetota. Microb. Genom. 12, 10.1099/mgen.0.001634 41944727 PMC13055916

[41] Hwang S., Lee N., Cho S., Palsson B. and Cho B. (2020) Repurposing modular polyketide synthases and non-ribosomal peptide synthetases for novel chemical biosynthesis. Front. Mol. Biosci. 7, 87 10.3389/fmolb.2020.00087 32500080 PMC7242659

[42] Bağcı C., Nuhamunada M., Goyat H., Ladanyi C., Sehnal L., Blin K. et al. (2025) BGC Atlas: a web resource for exploring the global chemical diversity encoded in bacterial genomes. Nucleic Acids Res. 53, D618–D624 10.1093/nar/gkae953 39470730 PMC11701567

[43] Paccagnella D., Bağcı C., Gavriilidou A. and Ziemert N. (2025) PanBGC: a pangenome-inspired framework for comparative analysis of biosynthetic gene clusters. ISME Commun. 5, ycaf225 10.1093/ismeco/ycaf225 41403705 PMC12704434

[44] Machushynets N., Elsayed S., Du C., Siegler M., de la Cruz M., Genilloud O. et al. (2022) Discovery of actinomycin L, a new member of the actinomycin family of antibiotics. Sci. Rep. 12, 21345 10.1038/s41598-022-06736-0 35181725 PMC8857259

[45] Thapa B., Huo C., Budhathoki R., Chaudhary P., Joshi S., Poudel P. et al. (2024) Metabolic comparison and molecular networking of antimicrobials *Streptomyces* species. Int. J. Mol. Sci. 25, 4193 10.3390/ijms25084193 38673777 PMC11050201

[46] Ferreira C., Alfaro C., Pirro V. and Cooks R.G. (2025) Desorption electrospray ionization mass spectrometry: advances in instrumentation, high-throughput analysis, and imaging applications. Anal. Methods 17, 436–455 10.1039/d5ay01323b 41117085

[47] Rodrigues J., Prova S., Moraes L. and Ifa D. (2018) Characterization and mapping of secondary metabolites of *Streptomyces* sp. from caatinga by desorption electrospray ionization mass spectrometry (DESI–MS). Anal. Bioanal. Chem. 410, 7135–7144 10.1007/s00216-018-1315-0 30196421

